# Devastating Wildfires and Mental Health: Major Depressive Disorder Prevalence and Associated Factors among Residents in Alberta and Nova Scotia, Canada

**DOI:** 10.3390/bs14030209

**Published:** 2024-03-05

**Authors:** Wanying Mao, Reham Shalaby, Belinda Agyapong, Gloria Obuobi-Donkor, Raquel Da Luz Dias, Vincent I. O. Agyapong

**Affiliations:** 1 Department of Psychiatry, University of Alberta, Edmonton, AB T6G 2H5, Canada; 2 Department of Psychiatry, Dalhousie University, Halifax, NS B3H 2E2, Canadaraquell.dias@nshealth.ca (R.D.L.D.); 3 QEII Health Sciences Centre, Department of Psychiatry, Faculty of Medicine and Dentistry, Dalhousie University, 5909 Veterans Memorial Lane, 8th Floor, Abbie J. Lane Memorial Building, Halifax, NS B3H 2E2, Canada

**Keywords:** wildfire, depression, predictors, natural disaster, mental health

## Abstract

***Background***: Since March 2023, hundreds of fires have burned from coast to coast throughout the country, placing Canada on track to have the worst wildfire season ever recorded. From East to West, provinces such as Quebec, Ontario, Nova Scotia, Alberta, and British Columbia have been particularly affected by large and uncontrollable wildfires. ***Objectives***: The objective of this study was to determine the prevalence of depression symptoms and predictors among residents living in extreme climate conditions during the Canadian wildfires of 2023 in Alberta and Nova Scotia and to update the literature with data related to those wildfires. ***Methods***: A cross-sectional quantitative survey was conducted in this study. REDCap was used to administer an online survey between 14 May and 23 June 2023. Through the Text4Hope program, participants subscribe to receive supportive SMS messages daily. As part of the initial welcome message, participants were invited to complete an online questionnaire, containing demographic information, wildfire-related information, and responses to the Patient Health Questionnaire-9 (PHQ-9) for depression assessment. SPSS version 25 was used to analyze the data. Descriptive, univariate, and multivariate regression analyses were employed. ***Results***: A total of 298 respondents completed the survey out of 1802 who self-subscribed to the Text4Hope program in Alberta and Nova Scotia and received a link to the online survey, producing a response rate of 16.54%. Most of the respondents were females (85.2%, 253), below 40 years of age (28.3%, 84), employed (63.6%, 189), and in a relationship (56.4%, 167). A historical depression diagnosis (OR = 3.15; 95% CI: 1.39–7.14) was a significant predictor of moderate to severe MDD in our study. The unemployed individuals were two times more likely to report moderate to severe symptoms of MDD than employed individuals (OR = 2.46; 95% CI: 1.06–5.67). Among the total sample population, the moderate to severe MDD prevalence was 50.4%, whereas it was 56.1% among those living in areas affected by wildfires. ***Conclusion***: Based on our study findings, unemployment and a history of depression diagnosis were independently significant risk factors associated with the developing moderate to severe MDD symptoms during wildfire disasters. Further research is required to identify robust predictors of mental health disorders in disaster survivors and provide appropriate interventions to the most vulnerable communities and individuals.

## 1. Introduction

Each year, about 8000 wildfires occur in Canada [1]. From May through September, wildfires are common in Canada’s forested and grassland regions, which can cause extensive damage and pose a threat to public health and property [2]. The Canadian provinces have been adversely affected by a series of wildfires that have been ongoing since March 2023, with increased intensity beginning in June. As the worst wildfire season in Canadian and North American history since the 2020 California wildfires, many provinces and territories have been affected, particularly Alberta and Nova Scotia [2,3,4,5,6]. Considering the severity of the wildfires in 2023, the Canadian Interagency Forest Fire Centre has declared it to be the worst in the nation’s history, exceeding the fire season of 1989, 1995, and 2014 [7]. The fires were exacerbated by prolonged drought conditions and high temperatures across much of the country, forcing 155,000 people to flee their homes and requiring unprecedented international assistance [8].

Besides destroying a community and property, natural disasters such as wildfires often cause numerous health conditions. Except for the possibility of bodily injury resulting from fire heat, smoke is equally dangerous due to the large amounts of carbon dioxide and carbon monoxide released into the atmosphere during combustion [9,10]. Acute health effects of wildfires include reduced lung function, chronic lung disease exacerbation, neurological impairment, and increased mortality [9,10,11]. In the long term, wildfires increase hypertension, gastrointestinal disorders, diabetes, COPD, and asthma exacerbations among survivors [12].

The World Health Organization reports that after a disaster, the prevalence of mental health problems will rise from 10 to 20% for mild to moderate difficulties and from 2 to 4% for severe difficulties. Aside from damaging physical conditions, wildfires can have a significant negative impact on the mental health of residents of affected areas [10]. In the aftermath of a wildfire, major depressive disorder (MDD) is one of the most studied and screened mental health conditions [10,13]. The American Psychiatric Association states that MDD is a serious medical illness that negatively impacts patients’ feelings, thoughts, and behaviors [14]. People suffering from depression may feel sad and/or lose interest in previous activities. This may lead to a variety of emotional, physical, and functional problems [14]. Several studies have found a significant increase in depression among children, adolescents, and adult populations following wildfires [15,16,17,18]. A scoping review of sixty studies found 4.9% to 54% of respondents reported depressive symptoms after wildfires, and the effects of wildfire on depression can last for 10 years [10,19]. Fort McMurray wildfire study results show fourfold and twofold increases in MDD prevalence among survivors six months and eighteen months after the fires, respectively, compared with the province’s general population [10,20,21,22]. In a similar vein, Bryant et al. found that three to four years after the Victorian Black Saturday bushfire, survivors reported higher rates of depression incidents [23]. During the three follow-up periods, depression rates were consistently around 10% in the high-impact group [23].

A lot more research has been conducted on the risk factors for depression in the adult population following a disaster like a wildfire compared to other mental health conditions. These predictors include sociodemographic characteristics (e.g., female gender) [10,20,21,23], pre-existing mental health diagnoses (e.g., depression) [10,20], trauma factors (e.g., witnessing or experiencing property loss) [18,20,21,23], post-trauma risk factors (e.g., inadequate family/social support) [10,20,21,23], and ongoing life stressors (e.g., poverty) [20,21,23]. A similar pattern of risk factors has been identified for depression in pediatric populations following wildfires, although the female gender has not consistently been statistically significant [15,16,24].

This study was part of the evaluation of the Text4Hope intervention, a service provided to people during crises [25]. In early 2023, while the Canadian wildfire was at its peak, we conducted a study to assess the impact of the wildfire on survivors’ mental health. Whilst there have been a few prior studies that have examined the prevalence and corelates of MDD post-wildfires, this study is unique because the prevalence and correlates of MDD were assessed during the wildfires. In addition, whilst previous studies have focused on individuals in one geographical location, this study is focused on residents of two provinces in the east and west of Canada, with participants selected at random from residents in both Alberta and Nova Scotia. We investigated the prevalence of MDD among respondents and analyzed the possible demographic, clinical, trauma-related, and other risk factors that could contribute to MDD development by using self-administered questionnaires. The study also aimed to improve understanding of the significance of recovery for mental health outcomes. Consequently, countries such as Canada that are particularly vulnerable to and consistently and significantly suffer a lot from natural disasters such as wildfire might be better equipped to support those who may experience mental health problems after disasters in the future.

## 2. Methodology

### 2.1. Study Setting

As part of two mental health support services, Text4Hope Alberta and Text4Hope Nova Scotia, this study was conducted in two Canadian provinces, Alberta and Nova Scotia. The Text4Hope program was initially developed by a team of academics and clinicians based in the Department of Psychiatry, University of Alberta, and Alberta Health Services during the pandemic of COVID-19. One week after the launch, the program received 32,805 subscriptions, and each day, the number of subscribers grows by the minute [25,26]. With the start of the 2023 Canadian wildfires, the Text4Hope program was contextualized and promoted to support residents of Alberta and Nova Scotia who had been impacted by the wildfires.

Located in Western Canada, Alberta is one of the three prairie provinces [27]. With a total area of 661,848 square kilometers and a population of 4,262,635 individuals, it is the fourth-largest province in terms of area and population [27,28]. Alberta declared a state of emergency on 6 May. As of 12 June 2023, the province could set a record for the number of areas burned by wildfires in 2023, with an estimated 1,400,021 hectares of land burned, exceeding the previous record of 1,357,000 hectares in 1981 [29,30]. Over 29,000 people have been evacuated from multiple communities as a result of evacuation orders [30].

Nova Scotia occupies a total area of 55,284 square kilometers, making it the second-smallest province in Canada. The province is one of the three Maritime provinces and one of the four Atlantic provinces, with a population of 1,037,782 [31]. In early June, the fire had grown to more than 235 square kilometers, making it the largest fire in the province’s history. Sixteen thousand people were forced to leave their homes as a result of this fire, and 151 homes as well as dozens of other structures were destroyed [32].

### 2.2. Study Design, Data Collection, and Ethical Consideration

A quantitative cross-sectional survey was conducted in this study. We collected data between 14 May and 23 June 2023 using a self-administered online questionnaire through the use of REDCap, which is an application designed for developing and managing online surveys and translational research databases [33]. Through the Text4Hope program, participants self-subscribe to receive daily supportive SMS messages by texting “HOPENS” or “HOPEAB” to 393939, depending on their province of residence [26]. Mental health therapists and members of our research have written messages crafted within the cognitive behavioral framework. The online software is programmed to deliver the messages at 9 a.m. each day [26]. Following the first message, respondents are invited to complete an online baseline survey capturing demographic information, information related to wildfires, and responses to the Patient Health Questionnaire-9 (PHQ-9) for depression assessment (Cronbach alphas of 0.86) [34,35]. It is entirely voluntary for subscribers to participate in this program, and they can opt out at any time by texting “STOP” to 393939 [26].

Patients were assessed for probable MDD by using the PHQ-9 questionnaire [34]. In addition to being an effective tool for the patient population, the literature also indicates that it is a validated tool for the screening of depressive symptoms among the general population. Based on the standard recommendation, the PHQ-9 score was calculated, and the depression criteria were met if 5 of the 9 items were checked for at least “more than half the days” and either item A or B was observable for at least “more than half the days”. A score ≥ 10 denotes moderate to severe depression. In terms of its reliability and validity, the tool appears to have sound psychometric properties [34,36]. The internal consistency of the PHQ-9 has been shown to be high. A study involving two different patient populations produced Cronbach alphas of 0.86 and 0.89. As standard self-report measures were used, there was no potential for bias in the collected data [34].

Participants were provided with information about the study, and completing the survey questions implied informed consent. The study received ethical approval from the Health Research Ethics Board of the University of Alberta (Pro00086163) and the Research Ethics Board at Nova Scotia Health (REB file #1028254).

### 2.3. Sample Size Estimation

In the context of a population of 5,232,018 (969,383 in Nova Scotia and 4,262,635 in Alberta) as of the 2021 census [27], a 95% confidence interval, and a margin of error of ±5%, a sample size of 385 persons was required for the estimation of the prevalence of MDD.

### 2.4. Statistical Analysis

The data were analyzed using SPSS Version 25 (IBM Corp 2011, Armonk, NY, USA) [37]. Descriptive statistics for demographic characteristics were reported in numbers and percentages. We presented descriptive statistics for demographics, clinical factors, and disaster-related factors versus living areas (if living in an area affected by wildfires). We classified the living area into two groups: those living in the Alberta/Nova Scotia region recently affected by the wildfire and those not living in the region.

With the use of chi-squared tests/Fisher’s exact, cross-sectional analyses were conducted to examine relationships, categorical variables, and the likelihood that respondents had MDD. The results were reported without inputting the missing data. Based on the univariate analysis, variables with a statistically significant relationship (*p* ≤ 0.05, two-tailed) or approaching significance (0.05 < *p* ≤ 0.1, two-tailed) to the likelihood of MDD were included in a logistic regression analysis. Before running logistic regression, a correlational analysis was conducted to identify any significant intercorrelations (Spearman’s correlation coefficient of 0.7 to 1.0 or 0.7 to 1.0) among the predictor variables. To determine the relationship between each component in the model and the likelihood of respondents presenting with likely MDD, the odds ratios and confidence intervals from the binary logistic regression analysis were examined.

## 3. Results

**Figure 1:**
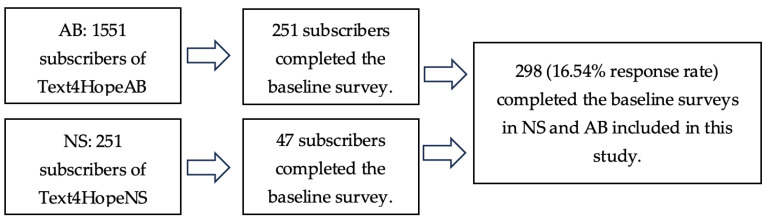
The Text4Hope survey flow chart.

According to the flow chart (Figure 1), between May 14 and June 23, 2023, the total number of subscribers to the Text4Hope program from Alberta is 1551, with 251 providing completed responses at baseline (response rate: 16.18%). From Nova Scotia, 251 subscribers subscribed to the service with 47 respondents providing completed responses at baseline (response rate 18.73%).

As illustrated in Table 1, descriptive characteristics of the sample (including sociodemographic information, living situation, disaster exposure, and clinical variables) are presented in relation to the respondents’ living areas. The association of these variables with moderate to severe depression and the descriptor variables is shown in Table 2. Table 3 shows the results of a logistic regression examining significant predictors of moderate to severe depression.

A total of 298 respondents completed the survey out of 1802 who accessed the online survey, producing a response rate of 16.54%. There were 112 (37.7%) respondents living in the disaster-affected area, 253 (85.2%) of the sample were female, 251 (84.2%) were from Alberta, 47 (15.8%) were from Nova Scotia, 248 (83.5%) are Caucasian, 213 (71.6%) are over 40 years old, 189 (63.6%) are employed, 246 (82.8%) have achieved post-secondary education, 167 (56.4%) are in a relationship, and 200 (67.3%) have their own homes. Regarding the related clinical data, 168 (56.4%) of our respondents had a history of depression diagnosis, 157 (52.7%) reported having an anxiety diagnosis, 233 (78.3%) reported taking medication for mental health concerns, and 151 (50.8%) respondents reported receiving mental health counseling in the past year. In terms of disaster-related and support information, 52 (46%) respondents reported receiving some to an absolute level of support from family, 19 (16.8%) from the Government of Alberta, and 6 (5.4%) from the Red Cross. In the total study population, the prevalence of moderate to severe MDD was 50.4%, while it was 56.1% among people living in the wildfire-affected area in our sample. A more detailed description of the characteristics of respondents can be found in Table 1.

### 3.1. Univariate Analysis

An overview of the results of the univariate analysis of the correlation between likely MDD in respondents and demographic, clinical, and wildfire-related experiences is shown in Table 2. A total of 15 variables were significantly associated with moderate to high symptoms of MDD. Among respondents under 40 years of age, moderate to severe MDD is more prevalent (58.1%) than among respondents over 40 years of age. Those with a high school education or less were more likely to present with moderate to high MDD (66.7%) than those with post-secondary education. The prevalence of MDD was higher (72.5%) among unemployed respondents than among employed or those who attended school. In comparison to those who owned their own home, those who rented apartments (62.7%) or lived with others (64.0%) were more likely to suffer from moderate to high symptoms of MDD. Those with a history of depression (63.8%), anxiety disorder (69.2%), personality disorder (72.2%), and ADHD (83.3%) prior to the wildfire in 2023 were more likely to present with symptoms of moderate to high MDD in comparison with those without these historical diagnoses. In addition, respondents who took antidepressants (63.1%), antipsychotics (73.7%), benzodiazepines (80.0%), mood stabilizers (69.6%), or sleep tablets (65.6%) had an increased risk of developing MDD compared with those who did not take any medication for mental health concerns.

### 3.2. Logistic Regression

For the purpose of determining the likelihood that respondents would demonstrate moderate to severe symptoms of MDD, we used a multivariable binomial logistic regression model, as shown in Table 3. We found 14 variables with significant (*p* ≤ 0.05) or near-significant *p* values (0.05 < *p* ≤ 0.1) (Table 2). Not on any medication for mental health concerns was dropped from the regression model as it was highly correlated with the variable of receiving antidepressants (rs > 0.7). Therefore, we ended up entering 14 variables into the logistic regression model to predict the likelihood of MDD.

The logistic model was statistically significant. Χ2 (df = 19, *n* = 250) = 51.11, *p* ≤ 0.00, showing that the model could distinguish respondents who had moderate to severe MDD symptoms from those who showed the lowest level of depression due to the wildfires. The model accounted for 18.5% (Cox and Snell R2) to 24.7% (Nagelkerke R2) of the variance, indicating the likelihood that respondents would present with MDD and accurately identified 71.2% of cases.

As shown in Table 3, compared to individuals without a history of MDD diagnosis, study participants who had a history of depression were more than three times more likely to suffer from moderate to severe MDD symptoms (OR = 3.15; 95% CI: 1.39–7.14). Furthermore, unemployed individuals were two times more likely to report moderate to severe symptoms of MDD than employed individuals (OR = 2.46; 95% CI: 1.06–5.67). The largest contribution to the model was provided by the historical depression diagnosis (Wald = 7.56).

## 4. Discussion

In this study, a self-administered survey was conducted during the 2023 Canadian wildfires in order to assess the prevalence and potential predictors of likely MDD among residents of Alberta and Nova Scotia provinces. Among respondents living in disaster-affected areas in both provinces, the overall prevalence of MDD was 56.1%.

It has been noted previously that the period of the 2023 Canadian wildfires was regarded as the worst wildfire season in North American history since the 2020 California wildfires [2,3,4,5,6], with the MDD prevalence much higher than the estimated lifetime MDD prevalence in Alberta (9.7%) and Canada (12.6%) [20]. Compared with the longitudinal study conducted in Fort McMurry (FMM), Canada, after the devastating wildfire of 2016, this study found a significantly higher prevalence of MDD. In the 6 months [22], 18 months [21], and five years following the FMM wildfires [10], MDD prevalence was 14.8%, 24.8%, and 45% among respondents, correspondingly. As well, this study has demonstrated a higher prevalence of MDD compared to previous surveys conducted on community-based wildfires. For example, MDD prevalence rates were reported at 12.9% and 33%, respectively, four years after the Victoria Black Saturday bushfire [23] and 3 months after the California firestorm in 2003 [18].

A historical depression diagnosis was the only clinical factor in our study that contributed to the development of MDD after the disaster. As indicated by this study, 63.8% of respondents who had previously been diagnosed with depression showed symptoms of MDD following the wildfire in 2023, which is three times more likely than those without a depression diagnosis. The findings of our study are in accordance with those of previous research that has examined patterns and predictors of depressive symptom trajectories following mass traumatic events [10,21,38,39,40,41,42]. Similar results were explained by resilience in the majority of studies. “Resilience” refers to the ability to continue functioning after a traumatic event and is characteristic of normal coping and adaptation to adversities of life [43,44]. It has been demonstrated to be a protective factor against the development of psychiatric disorders [45]. Depression may decrease a person’s resilience and could be one of the factors contributing to the non-functioning and poor-coping in people struggling with depression after a disaster. In one study, the association between trait resilience and depressive symptoms were examined among adolescent survivors of the Wenchuan earthquake in China [38]. The results indicated that resilience was negatively associated with depression, meaning that the more severe the survivor’s depression was, the less resilient they were [38]. Even though resilience is not the factor we are measuring in this study, a large body of published literature has investigated and depicted the significant relationship between resilience and mental health following disasters [10]. 

It is also possible to interpret our study results from the standpoint of depression relapse. Major depression is not a one-and-done type of condition. In most cases, it occurs more than once during a patient’s lifetime [46]. For many individuals, this is a chronic or lifelong illness, often accompanied by relapses or recurrences over the course of their lives [46]. Researchers have found that stressful life events and trauma are primarily responsible for depression relapses. Traumatic life events, such as the loss of a job, the death of a loved one, or experiencing a natural disaster, can easily trigger a new depression episode [46,47].

Among the demographic characteristics analyzed in this study, being unemployed was closely associated with the development of moderate to severe MDD after a wildfire, with the prevalence of depression rated at 72.5% among the unemployed population. The study’s result is consistent with the study we performed five years after the FMM wildfire, which showed that unemployed respondents were almost ten times more likely to present with likely MDD than employed respondents [10]. Research has long been conducted and documented in the literature indicating the relationship between unemployment and poor mental wellbeing [10,48,49,50,51,52,53,54]. An example is Eisenberg and Lazarsfeld’s 1938 review paper published after the Great Depression of the 1930s. In their study, they concluded that unemployment tends to lead to an increase in emotional instability [53]. A meta-analysis of 89 studies that examined the relationship between unemployment and mental illness revealed a prevalence of MDD of 24% [52], which is considerably higher than the prevalence of the disorder observed in studies conducted among the general population (between 7.2 and 12.9%) [52,55].

Multiple mechanisms may contribute to unemployment being associated with an increased risk of depression. As for the economic factor dimension, research has shown that reduced income is associated with a greater risk of depression and that depression is more prevalent among low-income individuals [52,56,57,58]. There is also the possibility that unemployed people are more likely to engage in unhealthy behavior and lifestyles, which may increase their risk of developing MDD [10,59,60]. Some other possible explanations include being unemployed causing anxiety about income loss and the possibility of a future decline in the standard of living, even if there is no material deprivation [53], and unemployment can lead to a drop in status among friends and family, which in turn can lead to stigma and the loss of self-esteem [61]. Moreover, it is typical for those who lose their jobs to lose contact with their work colleagues and their social networks to shrink. Personal wellbeing can decline as a result of a loss of engagement and “social capital” [62]. Nevertheless, the contrary is also supported by evidence. The study evaluating the prevalence and predictors of MDD in FMM during COVID-19 found that unemployment did not statistically significantly affect depression symptoms [63]. However, given the relatively low unemployment rate in FMM (6%) during the pandemic, as well as the wide confidence intervals, the author acknowledges that this lack of association should be interpreted cautiously and studies with larger sample sizes may be required for further clarification [63].

As a notable point, our study did not identify any significant associations between MDD development following the wildfire and some commonly recognized risk factors, such as female gender, young age, and inadequate social support, living in impacted areas, etc. It is possible that, unlike many similar studies that were conducted weeks or several months after a disaster, this study was conducted during the wildfire, and thus, the study may not be able to elicit some of the risk predictors of mental health conditions in the long term. However, other studies conducted in Alberta have found significant association between predictor factors such as gender and age and mental health symptoms following wildfire disasters [21,22]. Future studies with larger sample sizes are needed to better clarify the prevalence and predictors of MDD in relation to wildfires. In addition, in order to help develop further understanding of the temporal relationship between MDD symptoms onset and wildfires, future studies should be conducted at multiple time points during and after wildfires.

## 5. Limitations

Our study is not without limitations. In the first place, the sample size is relatively small; therefore, we may need to replicate our findings in larger-scale studies. Secondly, the wildfires may have affected internet connections in some specific areas, and some older individuals may have difficulty responding to online surveys. The majority of our study participants are young to middle-aged Caucasian females, which may limit the generalizability of the study results. Additionally, self-reported questionnaires, such as the PHQ-9 survey, have been used in place of a formal assessment by a clinician to assess those likely to suffer from MDD, which may undermine the validity of the study findings. Furthermore, the findings of this study may be limited by selection bias, as our respondents were Text4Hope subscribers who may have opted for the service to receive mental health support, thereby affecting the generalizability of the results. Lastly, with a sample size of 298 rather than the 385 we had expected, our estimations of the prevalence rates for MDD had a margin of error of ±5.68% at 95% confidence intervals, rather than the ±5% we had anticipated. The low response rate (16.54%) is another limitation of this study. Possible explanations include the fact that people with severe MDD might not opt to participate in the study. Additionally, many people living in disaster-affected areas might have experienced a challenging time in the aftermath of the disaster and are busy trying to get their lives back on track. Understandably, some suffering from this kind of situations may not show the potentials to respond to an online study survey. Although a low response rate does not necessarily affect the validity of the collected data [64], in order to maximize validity, it is still necessary to test for non-response effects and make corrections to the original data for future study. With the present study design, we sought to identify possible associated factors that may be predictive of MDD status. Nevertheless, longitudinal studies would be ideal for concluding cause–effect relationships.

In spite of the limitations noted above, this study contributes to the limited literature available in the field of mental health in relation to the recent Canadian wildfire. In addition, this study adds to the clinical and research evidence in the area of trauma and disasters.

## 6. Conclusions

This study examined the prevalence rates of MDD among residents in Alberta and Nova Scotia during the catastrophic Canadian wildfires of 2023. We also examined demographic, clinical, and other risk factors associated with likely MDD in the participants. In the disaster-affected areas, 56.1% of participants reported moderate to severe depression as a result of the wildfire. Unemployment and a previous diagnosis of MDD were significantly associated with the development of moderate to severe depression following the disaster.

Disasters do not just disrupt the quality of life but also impose a significant burden on mental health conditions for individuals and communities. For governments, it is noteworthy that the psychological distress of victims is just as prevalent as socioeconomic distress. The adverse mental health effects of a natural disaster can be ameliorated by providing effective interventions prior to, during, and after the disaster. Combining psychosocial education and clinical interventions is expected to provide better results through the integration of a variety of effective measures. To improve functionality and achieve a better quality of life for the entire community, it is necessary that researchers and policymakers utilize resources that examine and prevent developing mental disorders such as depression to the greatest extent, with a thorough understanding of the underlying causes of the degradation of mental well-being during disasters. Additionally, it is critical that the rehabilitation plan considers the cultural context of the community and the needs of its residents so that it can better facilitate the community’s ability to cope with future disasters holistically.

## Figures and Tables

**Table 1:** Distribution of sociodemographic, clinical characteristics, and wildfire-related items among the study participants.

Variables	Living in a Region of Nova Scotia/Alberta That Has Recently Been Impacted by the Wildfires		
	No (Total = 185) *n* (%) 62.3%	Yes (Total = 113) *n* (%) 37.7%	Total 297 *n* (%)
**Province**			
Nova Scotia	19 (10.3%)	28 (24.8%)	47 (15.8%)
Alberta	166 (89.7%)	85 (75.2%)	251(84.2%)
**Age**			
Median	50.00	50.50	
Mean (SD)	48.62 (14.05)	47.98 (13.79)	
**Age categories**			
≥60 year	48 (25.9%)	24 (21.4%)	72 (24.2%)
50–59	46 (24.9%)	34 (30.4%)	80 (26.9%)
40–49	39 (21.1%)	22 (19.6%)	61 (20.5%)
≤40 year	52 (28.1%)	32 (28.6%)	84 (28.3%)
**Gender**			
Male	25 (13.5%)	13 (11.6%)	38 (12.8%)
Female	156 (84.3%)	97 (86.6%)	253 (85.2%)
Other	4 (2.2%)	2 (1.8%)	6 (2.0%)
**Ethnicity**			
Caucasian	150 (81.1%)	98 (87.5%)	248 (83.5%)
Indigenous	12 (6.5%)	6 (5.4%)	18 (6.1%)
Asian	10 (5.4%)	1 (0.9%)	11 (3.7%)
Black/Hispanic	6 (3.2%)	3 (2.7%)	9 (3.0%)
Other	7 (3.8%)	4 (3.6%)	11 (3.7%)
**Education level**			
High School or Lower Education	34 (18.4%)	17 (15.2%)	51 (17.2%)
Post-secondary Education	151 (81.6%)	95 (84.8%)	246 (82.8%)
**Relationship status**			
Married/Partnered/Common Law/Cohabiting	106 (57.6%)	61 (54.5%)	167 (56.4%)
Single	48 (26.1%)	27 (24.1%)	75 (25.3%)
Separated or Divorced	22 (12.0%)	16 (14.3%)	38 (12.8%)
Widowed	5 (2.7%)	7 (6.3%)	12 (4.1%)
Other	3 (1.6%)	1 (0.9%)	4 (1.4%)
**Employment status**			
Employed	116 (62.7%)	73 (65.2%)	189 (63.6%)
Unemployed	30 (16.2%)	18 (16.1%)	48 (16.2%)
Student	7 (3.8%)	5 (4.5%)	12 (4.0%)
Retired	32 (17.3%)	16 (14.3%)	48 (16.2%)
Housing status			
Own home	121 (65.4%)	79 (70.5%)	200 (67.3%)
Renting accommodation	44 (23.8%)	20 (17.9%)	64 (21.5%)
Live with family or friend	20 (10.8%)	13 (11.6%)	33 (11.1%)
History of having mental health diagnosis from a health professional *			
Depression	107 (57.8%)	61 (54.0%)	168 (56.4%)
Bipolar Disorder	10 (5.4%)	6 (5.3%)	16 (5.4%)
Anxiety	98 (53.0%)	59 (52.2%)	157 (52.7%)
Alcohol abuse	11 (5.9%)	1 (0.9%)	12 (4.0%)
Drug abuse	8 (4.3%)	3 (2.7%)	11 (3.7%)
Schizophrenia	2 (1.1%)	1 (0.9%)	3 (1.0%)
Personality Disorder	10 (5.4%)	10 (8.8%)	20 (6.7%)
PTSD/OCD	9 (4.9%)	10 (8.8%)	19 (6.4%)
ADHD	7 (3.8%)	7 (6.2%)	14 (4.7%)
Other	2 (1.1%)	2 (1.8%)	4 (1.3%)
No mental health diagnosis	35 (18.9%)	28 (24.8%)	63 (21.1%)
History of receiving psychotropic medications *			
Antidepressants	76 (41.1%)	40 (35.4%)	116 (38.9%)
Antipsychotics	12 (6.5%)	9 (8.0%)	21 (7.0%)
Benzodiazepines	8 (4.3%)	8 (7.1%)	16 (5.4%)
Mood stabilizers	16 (8.6%)	11 (9.7%)	27 (9.1%)
Sleeping tablets	18 (9.7%)	15 (13.3%)	33 (11.1%)
Stimulants for ADHD	6 (3.2%)	4 (3.5%)	10 (3.4%)
Other	10 (5.4%)	0 (0.0%)	10 (3.4%)
On no psychotropic medication	87 (47.0%)	61 (54.0%)	148 (49.7%)
**Having received MH counselling in the past year**			
No	88 (47.6%)	97 (52.4%)	146 (49.2%)
Yes	58 (51.8%)	54 (48.2%)	151 (50.8%)
**Having a wildfire evacuation order issued for the subscriber’s area of residence**			
Yes	N/A	25 (22.1%)	113 (100%)
No		88 (77.9%)	
**Having had to evacuate from your home due to the recent wildfires in AB/NS**			
No	N/A	88 (77.9%)	113 (100%)
Yes		25 (22.1%)	
Have you lost any property because of the wildfire?			
No	N/A	109 (96.5%)	113 (100%)
Yes		4 (3.5%)	
If you lost any property, what kind of property that was lost *			
Home	N/A	3 (60%)	4 (100%)
Car		2 (40%)	
Farm		0 (0%)	
**Having received support from family and friends in relation to the recent wildfire**			
Absolute support	N/A	28 (24.8%)	113 (100%)
Some support		24 (21.2%)	
Only limited support		18 (15.9%)	
Not at all		43 (38.1%)	
**Having received support from the government of AB/NS in relation to the recent wildfire**			
Absolute support	N/A	6 (5.3%)	6 (5.3%)
Some support		13 (11.5%)	13 (11.5%)
Only limited support		8 (7.1%)	8 (7.1%)
Not at all		86 (76.1%)	86 (76.1%)
**Having received support from Red Cross in relation to the recent wildfire**			
Absolute support	N/A	0	112 (100%)
Some support		6 (5.4%)	
Only limited support		4 (3.6%)	
Not at all		102 (91.1%)	
**Frequency of watching television images about the devastation caused by the recent wildfires in AB/NS**			
Daily	64 (34.6%)	41 (36.6%)	105 (35.4%)
About every other day	34 (18.4%)	25 (22.3%)	59 (19.9%)
About once a week	23 (12.4%)	8 (7.1%)	31 (10.4%)
Less than once a week	22 (11.9%)	16 (14.3%)	38 (12.8%)
Haven’t watched TV images of the devastation	42 (22.7%)	22 (19.6%)	64 (21.5%)
**Having called the Mental Health Criss line in relation to the recent wildfires in AB/NS**			
No	183 (98.9%)	110 (97.3%)	293 (98.3%)
Yes	2 (1.1%)	3 (2.7%)	5 (1.7%)
**Likely depression**			
Non to mild depression	81 (53.3%)	43 (43.9%)	124 (49.6%)
Moderate to severe depression	71 (46.7%)	55 (56.1%)	126 (50.4%)

* Multiple response question.

**Table 2:** Association analysis of demographic, clinical, and wildfire characteristics against likely depression.

Variables	Moderate to Severe Depression		
	*n* (%)	Chi ^2^ (DF)/Fisher’s Exact	*p*-Value
**Province**			
NS	21 (48.8%)	0.05 (1)	0.82
AB	105 (50.7%)		
**Age categories**			
≥60 year	24 (39.3%)	6.77 (3)	0.08
50–59	35 (56.5%)		
40–49	24 (44.4%)		
≤40 year	43 (58.9%)		
**Gender**			
Male	14 (48.3%)	*	0.82
Female	108 (50.2%)		
Other	4 (66.7%)		
**Ethnicity**			
Caucasian	102 (48.6%)	*	0.15
Indigenous	11 (68.8%)		
Asian	4 (40.0%)		
Black/Hispanic	3 (42.9%)		
Other	6 (85.7%)		
**Education level**			
High School or Lower Education	24 (66.7%)	4.45 (1)	0.04
Post-secondary Education	102 (47.7%)		
**Relationship status**			
Married/Partnered/Common Law/Cohabiting	64 (45.1%)	*	0.11
Single	36 (59.0%)		
Separated or Divorced	17 (50.0%)		
Widowed	8 (80.0%)		
Other	1 (33.3%)		
**Employment status**			
Employed	77 (47.0%)	*	0.01
Unemployed	29 (72.5%)		
Student	6 (66.7%)		
Retired	14 (37.8%)		
**Housing status**			
Own home	73 (44.0%)	8.17 (2)	0.02
Renting accommodation	37 (62.7%)		
Live with family or friend	16 (64.0%)		
History of having mental health diagnosis from a health professional *			
Depression	95 (63.8%)	26.33 (1)	0.00
Bipolar Disorder	9 (69.2%)	1.95 (1)	0.16
Anxiety	83 (58.5%)	8.52 (1)	0.00
Alcohol abuse	7 (58.3%)	0.32 (1)	0.57
Drug abuse	5 (45.5%)	0.11 (1)	0.74
Schizophrenia	0 (0.0%)	*	0.12
Personality Disorder	13 (72.2%)	3.70 (1)	0.06
PTSD/OCD	12 (66.7%)	2.05 (1)	0.15
ADHD	10 (83.3%)	5.47 (1)	0.02
Other	3 (75.0%)	*	0.62
No mental health diagnosis	17 (28.8%)	14.40 (1)	0.00
History of receiving psychotropic medications *			
Antidepressants	65 (63.1%)	11.31 (1)	0.00
Antipsychotics	14 (73.7%)	4.46 (1)	0.04
Benzodiazepines	12 (80.0%)	5.59 (1)	0.02
Mood stabilizers	16 (69.6%)	3.72 (1)	0.05
Sleeping tablets	21 (65.6%)	3.40 (1)	0.07
Stimulants for ADHD	3 (42.9%)	*	0.72
Other	5 (55.6%)	*	1.0
On no psychotropic medication	49 (40.5%)	9.20 (1)	0.00
**Having received MH counselling in the past year**			
No	54 (46.6%)	1.28 (1)	0.26
Yes	72 (53.7%)		
**Living in a region of AB/NS that has recently been impacted by the wildfires**			
No	71 (46.7%)	2.11 (1)	0.15
Yes	55 (56.1%)		
**Having a wildfire evacuation order issued for the subscriber’s area of residence**			
Yes	15 (62.5%)	*	0.33
No	38 (56.7%)		
Not applicable	2 (28.6%)		
**Having had to evacuate from your home due to the recent wildfires in AB/NS**			
No	41 (53.2%)	1.20 (1)	0.27
Yes	14 (66.7%)		
**Have you lost any property because of the wildfire?**			
No	53 (55.2%)	*	0.50
Yes	2 (100.0%)		
**Kind of property that was lost**			
Home	2 (100%)	*	0.50
Car	0 (0%)	-	-
Farm	0 (0%)	-	-
**Having received support from family and friends in relation to the recent wildfire**			
Some to absolute support	21 (50.0%)	1.12 (1)	0.29
Limited to no support	34 (60.7%)		
**Having received support from the government of AB/NS in relation to the recent wildfire**			
Some to absolute support	8 (53.3%)	0.06 (1)	0.81
Limited to no support	47 (56.6%)		
**Having received support from Red Cross in relation to the recent wildfire**			
Some to absolute support	4 (80.0%)	*	0.38
Limited to no support	51 (54.8%)		
**Frequency of watching television images about the devastation caused by the recent wildfires in AB/NS**			
Daily	41 (47.7%)	0.79 (4)	0.94
About every other day	27 (55.1%)		
About once a week	12 (48.0%)		
Less than once a week	16 (50.0%)		
Haven’t watched TV images of the devastation	30 (51.7%)		
**Having called the Mental Health Criss line in relation to the recent wildfires in AB/NS**			
No	125 (50.6%)	*	0.62
Yes	1 (33.3%)		

DF: degree of freedom. * Fisher’s Exact

**Table 3:** Logistic regression results of study respondents to present with likely depression.

		B	S.E.	Wald	df	Sig.	Exp(B)	95% C.I.for EXP(B)	
								Lower	Upper
**Age**	≥60 year			2.148	3	0.542			
	50–59 year	0.484	0.463	1.090	1	0.296	1.622	0.654	4.023
	40–49 year	−0.099	0.504	0.038	1	0.845	0.906	0.337	2.433
	<40 year	0.145	0.485	0.089	1	0.765	1.156	0.447	2.990
**Employment status**	Employed			5.664	3	0.129			
	Unemployed	0.899	0.427	4.429	1	0.035	2.457	1.064	5.674
	Student	0.461	0.838	0.303	1	0.582	1.586	0.307	8.196
	Retired	−0.358	0.518	0.476	1	0.490	0.699	0.253	1.932
**Education**	Post-secondary Education	−0.527	0.434	1.478	1	0.224	0.590	0.252	1.381
**Housing status**	Own Home			1.636	2	0.441			
	Rented Accommodation	0.335	0.378	0.789	1	0.375	1.398	0.667	2.931
	Live with Family or Friends	0.576	0.518	1.237	1	0.266	1.779	0.645	4.912
**Previous mental health diagnosis**	Depression								
	Yes	1.147	0.417	7.558	1	0.006	3.150	1.390	7.138
	Anxiety								
	Yes	0.060	0.374	0.026	1	0.872	1.062	0.510	2.210
	Personality Disorder								
	Yes	−0.131	0.697	0.035	1	0.851	0.877	0.224	3.439
	ADHD								
	Yes	1.232	0.858	2.060	1	0.151	3.427	0.637	18.424
	Received no mental health diagnosis								
	Yes	0.284	0.545	0.271	1	0.603	1.328	0.456	3.867
**Are you on any of the following medications for a mental health concern?**	Antidepressants								
	Yes	0.260	0.339	0.587	1	0.443	1.297	0.667	2.519
	Antipsychotics								
	Yes	0.453	0.760	0.355	1	0.551	1.572	0.355	6.968
	Benzodiazepines								
	Yes	0.933	0.722	1.667	1	0.197	2.541	0.617	10.470
	Mood Stabilizers								
	Yes	0.068	0.640	0.011	1	0.916	1.070	0.305	3.755
	Sleeping Tablets								
	Yes	0.292	0.475	0.377	1	0.539	1.338	0.528	3.395
**Constant**	−0.967	0.677	2.038	1	0.153	0.380			

## Data Availability

The data that support the findings of this study are available from the corresponding author, Vincent Agyapong, upon reasonable request.
